# Genetic Diversity of Dahongjun, the Commercially Important “Big Red Mushroom” from Southern China

**DOI:** 10.1371/journal.pone.0010684

**Published:** 2010-05-18

**Authors:** Mochan Li, Junfeng Liang, Yanchun Li, Bang Feng, Zhu-Liang Yang, Timothy Y. James, Jianping Xu

**Affiliations:** 1 Department of Biology, McMaster University, Hamilton, Ontario, Canada; 2 Research Institute of Tropical Forestry, Chinese Academy of Forestry, Guangzhou, People's Republic of China; 3 Key Laboratory of Biodiversity and Biogeography, Kunming Institute of Botany, Chinese Academy of Sciences, Kunming, People's Republic of China; McGill University, Canada

## Abstract

**Background:**

In southern China, a wild ectomycorrhizal mushroom commonly called “Dahongjun” or “Big Red Mushroom” by the local residents, has been harvested, consumed, and/or exported as an exotic food for many years. Although ecologically and economically important, very little is known about this mushroom, including its diversity and population structure.

**Methodology and Principal Findings:**

In this study, we analyzed 122 samples from five local populations representing the known distribution ranges of this mushroom in southern China. We investigated the genetic diversity and geographic structure of this mushroom using sequences from four DNA fragments. Our analyses identified that this mushroom contained at least three divergent lineages: one corresponds to a recently described species *Russula griseocarnosa* from southern China and the remaining two likely represent two novel species. While these lineages were prominently structured geographically based on ITS sequences, evidence for ancient and/or recent gene flow was also identified within individual lineages. In addition, a local population from Ailaoshan in central Yunnan Province where 85 of our 122 specimens came from showed clear evidence of recombination.

**Conclusion and Significance:**

The ectomycorrhizal mushroom “Dahongjun” from southern China is a species complex with at least three divergent lineages. These lineages are largely geographically structured and there is evidence for recombination in nature. Our results indicate mature Dahongjun mushrooms with abundant basidiospores are important for the reproduction of this mushroom in nature and that individual populations of this species should be managed separately.

## Introduction

Fungi are important constituents of the global biosphere. In forest ecosystems, many fungi form symbiotic associations with plant roots, establishing a structure called mycorrhiza. Over 90% of land plants form mycorrhizal associations with fungi. These fungi help plants obtain essential minerals and water from the soil and can contribute to plants' disease resistance and drought tolerance [1]. While most of the fungal materials are subterranean and are not typically observed by the naked eye, some mycorrhizal fungi, especially those belonging to Basidiomycota, are easily noticeable during certain times of the year because they produce conspicuous fruiting bodies (mushrooms). Some of these mushrooms are collected as a source of exotic and highly - prized food for humans.

The mushroom genus *Russula* (Russulaceae; Russulales; Agaricomycetes; Basidiomycota) is a highly diverse group of ectomycorrhizal (EcM) fungi. Globally, about 750 species have been identified and characterized so far in this genus [2]. The genus is considered important both economically and ecologically. Ecologically, as EcM fungi, they promote healthy plant growth by delivering water and minerals, and by enhancing stress tolerance. In return, they obtain carbohydrates from their host plants. This symbiosis likely plays a critical role for maintaining biodiversity and geochemical cycling in forest ecosystems [3]. In typical ecosystems, *Russula* constitutes a significant amount of the EcM biomass, and its distribution ranges from the tropics to subtropics, temperate regions, and even arctic zones. It has been shown that *Russula* is one of the most dominant EcM fungi in forest EcM communities in terms of both frequency and abundance [4]. In terms of edibility, only a few *Russula* species, such as those in the *R. emetica* group and *R. subnigricans* Hongo, are poisonous. Many species are excellent food sources for human consumption, including *R. cyanoxantha* (Schaeff.) Fr., *R. virescens* (Schaeff.) Fr., and “Dahongjun” in this study [5].

The genus *Russula* is characterized by several easily identifiable features: the brittle consistency of the fleshy basidiocarp due to the presence of sphaerocysts, the lack of latex in the basidiocarp, and basidiospores with amyloid incrustations [6]–[7]. However, distinguishing individual species within this genus is often very difficult due to the large number of closely related species, extensive phenotypic plasticity among strains within species, and the lack of macro-morphological features to separate them. Similar problems also exist for many other groups of fungi. Consequently, there is an increasing use of molecular information to help define and identify new species or separate groups of morphologically indistinguishable species. At present, most studies on *Russula* infrageneric classification and population biology have been carried out using European and North American samples [8]–[13]. The diversity and genetic structure of *Russula* species in other parts of the world remain poorly understood.

Due to its highly variable climate and diverse topography, southern (especially southwestern) China is ranked as one of the world's 34 biodiversity hotspots [14]. For example, about 600 out of 2,000 edible fungal species worldwide occur in Yunnan Province in southwestern China [15]. Some of the economically important mushrooms from this region include *Boletus edulis* sensu lato, *Thelephora ganbajun* M. Zang, *Tricholoma matsutake* (S. Ito & S. Imai) Singer, and *Russula* spp. [16]–[20]. Indeed, large populations of *Russula* are found throughout Yunnan Province. Many *Russula* species are harvested and sold at local markets or exported for human consumption. Among these *Russula* mushrooms, one called ‘Dahongjun’ [“Big Red Mushroom” by indigenous people in Yunnan] is probably the most prominent. This mushroom has been harvested and traded in local, national and international markets for over 20 years. Like many other pricey gourmet wild mushrooms such as matsutake, ganbajun and chanterelles, *Russula* cannot be artificially cultivated because of their dependency on a living plant host. Therefore natural populations in the forests are the only source for the market.

Like many wild mushrooms in southern China, accurate official statistics of the market are not available for Dahongjun. However, our personal contacts with mushroom hunters and traders over the last few years indicated that collecting this mushroom in June and July each year often provides up to half of a household's annual income for many people in southern and central Yunnan. During these months, fresh Dahongjun may be sold for US$8.00/kg and dried products are often sold for US$50-80/kg. However, the significant profits, uncontrolled harvesting practices in recent years, disturbances of the forest ecosystem, and loss of habitats could threaten the wild populations of Dahongjun in the regions. To help sustain its natural populations and design effective conservation and utilization strategies, it is important to understand the populations of this mushroom in their native distribution range.

Historically, mushroom enthusiasts and mycologists have regarded Dahongjun in southern China as *R. vinosa* Lindblad, which was originally described in Europe [6], [12], [21]. The reliability of this identification for Chinese *R. vinosa* is dubious as no formal taxonomic investigation of this species has been conducted and there is little molecular information for Chinese *Russula* in any public databases. Recently, a population of Dahongjun from southern Yunnan was found to have sequences at the internal transcribed spacer regions (ITS) very different from those of the typical *R. vinosa* from Europe [12], [22], and this group was recently described as a new species *R. griseocarnosa* X. H. Wang et al. [20].

The objective of the current study is to investigate the genetic diversity and relationships among strains and populations of Dahongjun from their main distribution regions in southern China and to examine their phylogenetic relationships with other closely related and known *Russula* species. The DNA sequences at the ITS region were obtained for all 122 mushroom samples from five local populations in three geographic regions. Since the ITS is repeated within the nuclear ribosomal gene cluster, the polymorphisms observed for ITS could be due to variation among repeats on the same chromosome and/or variation between sequences on the two different chromosomes in a typical dikaryotic mushroom. To minimize the complications related to interpreting ITS sequence polymorphisms, a sub-set of 43 isolates were further analyzed using portions of three other genes: the nuclear large subunit of the ribosomal RNA gene (nucLSU rRNA), the mitochondrial small subunit of the ribosomal RNA gene (mtSSU rRNA), and the second largest subunit of the nuclear RNA polymerase enzyme II (RPB2). It should be noted that the nucLSU rRNA gene is located within the same gene cluster as the ITS and as a result, they are not completely independent. In addition, both the nucLSU rRNA and mtSSU rRNA genes are highly conserved, typically exhibiting limited or no variation among strains within the same species. Despite these drawbacks, these three gene fragments were chosen here because they have been found capable of distinguishing related fungal species and that there are well-established primers in public databases to amplify these genes for this group of organisms [6], [20]–[22]. The patterns of sequence variation within and among strains and populations were analyzed for these four genes using phylogenetic and population genetic methods. Our results suggest that the Dahongjun samples analyzed here from southern China includes at least three divergent lineages with each lineage containing samples from the two to three geographic regions.

## Results

### ITS sequence variation

**Figure 1:**
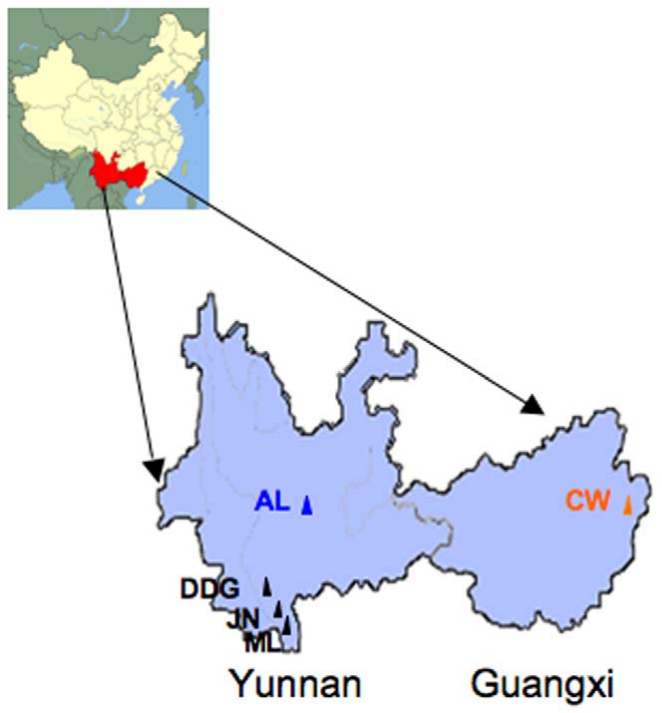
Dahongjun sample collection sites in southern China. Yunnan and Guangxi provinces are highlighted in red. Dadugang (DDG), Jinuo(JN) and Mengla (ML) in southern Yunnan region are marked as black triangles; Ailaoshan (AL) in central Yunnan is marked as a blue triangle, and Cangwu (CW) in eastern Guangxi region is marked as a yellow triangle.

A total of 122 Dahongjun specimens were collected from five geographic locations distributed in three regions: central Yunnan, southern Yunnan, and eastern Guangxi (Figure 1; Table 1). All specimens were successfully amplified using the ITS1 and ITS4 primer pair, and the resulting PCR products were sequenced in both directions (GenBank accession numbers FJ613896- FJ614017). These sequences were aligned using Clustal_X version 2 [23] and analyzed using PAUP [24]. Among the 683 aligned nucleotide sites, 82 were variable with 54 showing single base substitutions and 28 showing insertion/deletion (indels) (Table S1). The 28 indel sites were in 13 tracks with three containing three or more consecutive nucleotides. All the indels were found between strains and none was found within any of the 122 strains (Table S1). As a result, all our ITS sequence chromatograms were unambiguous from both directions.

**Table 1:** Geographic distribution and ITS sequence diversity of Dahongjun samples collected from Yunnan and Guangxi provinces in southern China.

Region	County/Community	Geographic coordinates	Sample size	ITS genotype (# isolates in each genotype)	Genotype diversity^1^
Central Yunnan	Ailaoshan (AL)	N24.32; E101.01	85	1(2); 2(4); 3(1); 4(1); 5(1); 6(1); 7(2); 8(1); 9(1); 10(1); 11(2); 12(1); 13(3); 14(1); 15(9); 16(1); 17(1); 18(1); 19(1); 20(16); 21(2) 22(1); 23(1); 24(1); 25(5); 26(1); 27 (4); 28(5); 29(1); 30 (2); 31(1); 32(2); 33(1); 34(4); 35(1); 36(1); 37(1)	0.945
Southern Yunnan	Mengla (ML)	N21.28; E101.35	9	38(1); 39(1); 41(1); 42(1); 43(1); 44(1); 45(1); 46(1); 54(1)	1.000
	Jinuo (JN)	N22.01; E101.03	8	39(1); 43(1); 49(1); 50(1); 47(2); 48(1); 51(1)	0.964
	Dadugang (DDG)	N22.20; E100.58	11	38(2); 40(2); 49(1); 50(2); 51(1); 55 (1); 56(1); 57(1)	0.945
Eastern Guangxi	Cangwu (CW)	N23.29; E111.18	9	58(2); 53(1); 52(1); 59(1); 60(1); 61(1); 62(1); 63(1)	0.972

1, Genotypic diversity is defined as the probability that two individuals taken at random have different genotypes. It's calculated as (1-∑p_i_^2^)•n/(n-1), where p_i_ is the frequency of the *i*th genotype and n is the number of individuals in the sample.

**Figure 2:**
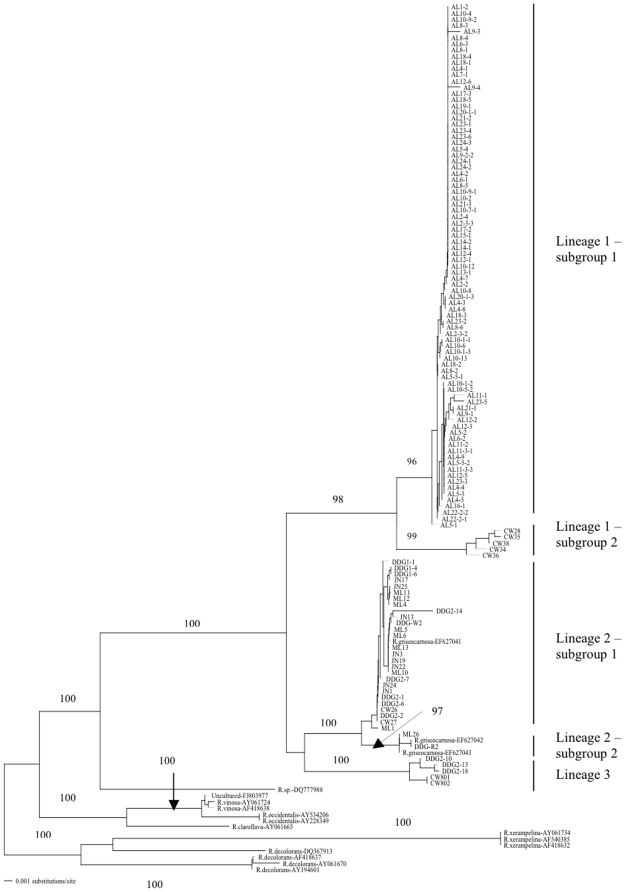
Maximum parsimony tree based on ITS sequences from 122 isolates of Dahongjun from southern China. Each strain label contains its geographic location information (AL: Ailaoshan, central Yunnan; CW: Cangwu, eastern Guangxi; DDG: Dadugang, southern Yunnan; JN: Jinuo, southern Yunnan; and ML: Mengla, southern Yunnan), followed by a field isolation number. Numbers along branches are bootstrap values greater than 90% obtained from 1000 replicates. Reference sequences contain species identification (when available) to to those in the database, followed the specific GenBank accession number. Sequences of non-Dahongjun samples are used as outgroups. Tree length = 368, Consistency index = 0.766, Retention index = 0.943.

Our analyses of these sequences suggested that our samples contained at least three phylogenetically distinct lineages (Figure 2; Table 1). The first lineage (Lineage 1) contained 85 strains from the Ailaoshan (AL) region in central Yunnan and 5 strains from Cangwu (CW) in eastern Guangxi (GX). This lineage contained a total of 42 ITS genotypes with 37 from AL and 5 from CW. The most common ITS genotype in the AL population was shared by 16 specimens and 25 genotypes from AL were represented by one isolate each. The second group (Lineage 2) contained 27 isolates, including 9 from Mengla (ML), 8 from Jinuo (JN), 8 from Dadugang (DDG), and 2 from CW. Among the 27 strains in Lineage 2, 17 ITS genotypes were identified. The most common genotype in Lineage 2 was shared by 3 isolates and 10 genotypes were each found in only one isolate. The third group (Lineage 3) contained 3 strains from DDG and 2 strains from CW. The three specimens from DDG each had a different ITS genotypes while the two specimens from CW shared a ITS genotype. The unique ITS genotype information and the distribution of ITS genotypes within and among the 5 local populations are presented in Table 1 and Table S1.

The sequence comparisons showed two notable stretches of sequence differences within the ITS2 region among the three lineages. Specifically, the first stretch includes a 3 bp-5 bp insertion/deletion in a poly-T region, with the insertions present among strains only in Lineage 2. The second stretch of sequence is an 18 bp insertion/deletion, with the deletion present among strains only in Lineage 3. Overall, we identified a total of 63 ITS sequence types and these sequence types differed between 1 and 90 nucleotide sites (out of 683 aligned nucleotides Tables S1 and S2). The numbers of total segregating sites, fixed and shared polymorphic sites within and between individual lineages are summarized in Table 2.

**Table 2:** ITS sequence divergence within and between phylogenetic lineages 1, 2, and 3 as indicated in Figure 2.

Pairwise lineage comparisons	No. of segregating sites	Fixed differences	Polym. 1 Monom. 2	Polym. 2 Monom. 1	Shared polymorphsims
Lineage 1- Lineage 2	63	21	25	15	2
Lineage 1- Lineage 3	77	47	21	8	1
Lineage 2- Lineage 3	54	37	11	4	2

Listed in the table are the numbers of total segregating sites, and fixed and shared polymorphic sites within and between lineages. The number of sites that are polymorphic found in the first group (or second group) but monomorphic in the second group (or first group) was also presented.

### Comparisons between our ITS sequences and those closely related from GenBank

ITS sequences representing the 63 ITS genotypes identified above were used as queries to identify similar sequences in GenBank through BLAST searches. We retrieved all sequences with a comparable length to ours and that showed an overall sequence identity ≥90% to our Dahongjun sequences. To compare the relative divergence of our sequences among each other and with those within and between other closely related species, we also included multiple ITS sequences from within several other *Russula* species whenever possible. In total, 17 reference sequences were retrieved from GenBank for comparisons with our sequences. These included three ITS sequences of *R. griseocarnosa* recently identified based on three specimens of Dahongjun in southern Yunnan [20] and 12 ITS sequences from GenBank representing 5 closely related species in *Russula* (*R. vinosa*, *R. claroflava*, *R. occidentalis*, *R. decolorans*, *R. xerampelina*; Supplemental Figure S1). In addition, one GenBank sequence obtained directly from an environmental DNA sample (FJ803977) and one mushroom not identified to the species level but was indicated as belonging to *Russula sp.* (DQ777988) also met our criteria (i.e. a comparable sequence length and ≥90% sequence identity to our sequences) and both were included in our analyses. The environmental sequence (FJ803977) differed at two and four nucleotide sites from the two known *R. vinosa* sequences, AY061724 and AF418638, respectively. We believe FJ803977 most likely belonged to *R. vinosa*.

The joint analyses of our sequences and those from the GenBank confirmed the distinctiveness of the three lineages within Dahongjun from southern China. One lineage (Lineage 2) corresponded to the known species, *R. griseocarnosa* recently identified from southern Yunnan [20]. The separation of these three lineages was supported by 98%, 100% and 100% bootstrap values. Similarly, the separation of Dahongjun from a closely related species *R. vinosa* had 100% bootstrap support, including large phylogenetic distances among them (Figure 2). Overall, the divergences among the three lineages identified here were comparable or greater than those among the closely related known *Russula* species (Figure 2).

### Phylogeographic pattern of ITS sequence variation

Interestingly, as shown in Figure 2 and mentioned above, each of the three lineages contained strains from both Yunnan and Guangxi provinces. However, within two of the three lineages, the samples from these two provinces seemed to cluster separately (Table 1 and Figure 2). Specifically, in Lineage 1, samples from AL and CW were partitioned into two separate subgroups. Similarly, in Lineage 3, the samples from DDG and CW were partitioned into two groups. A different pattern was seen in Lineage 2. Here, sixteen genotypes from ML, JN, DDG, CW and one genotype of *R. griseocarnosa* (also from DDG) were clustered as subgroup 1. Subgroup 2 within Lineage 2 included four specimens from southern Yunnan, with one from ML, one from DDG, and two isolates of *R. griseocarnosa* from Xishuangbana in southern Yunnan [20; Table 1; Figure 2].

### Multiple gene sequence analyses support the existence of three divergent lineages

**Figure 3:**
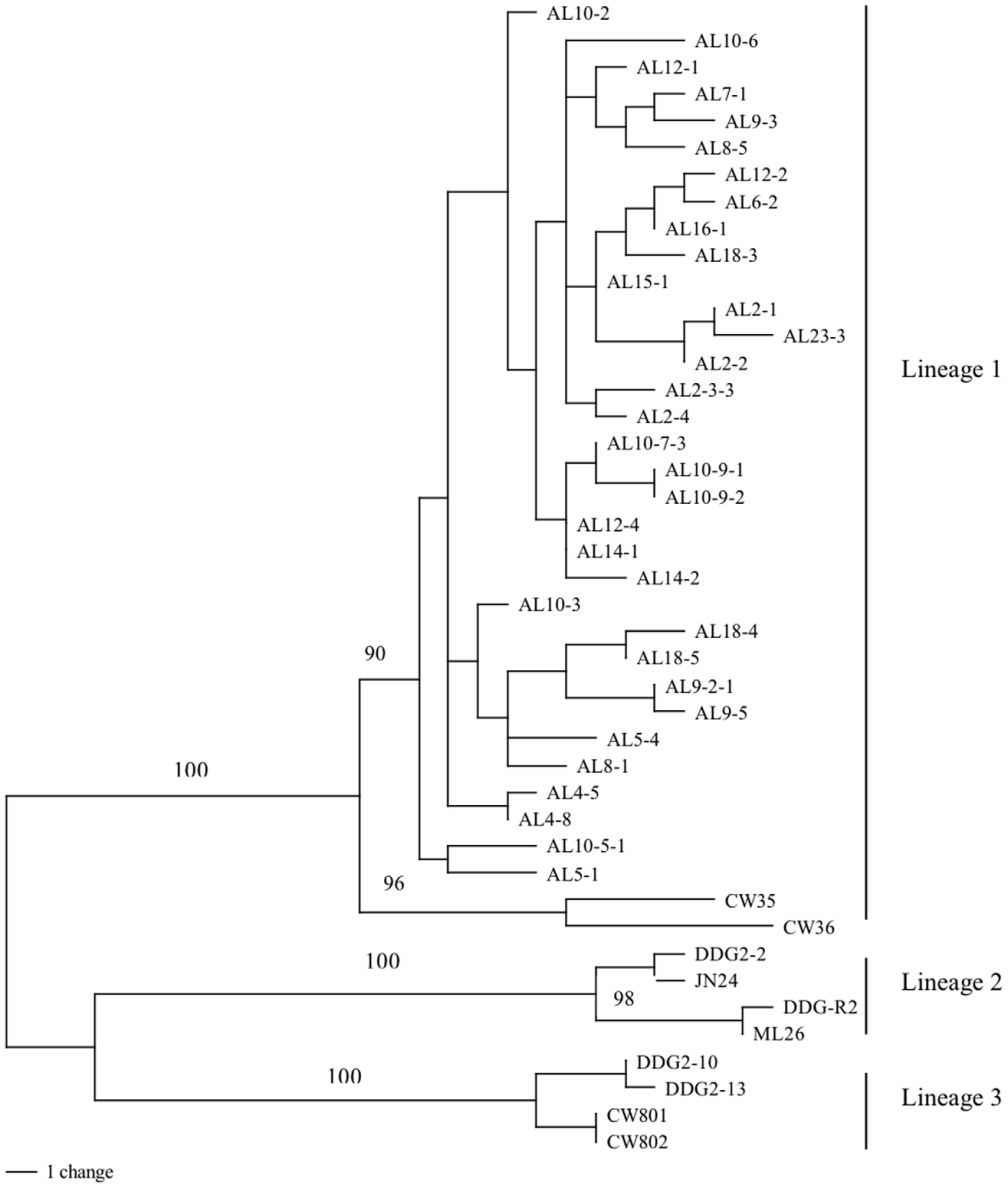
Maximum parsimony tree based on the concatenated sequences of four DNA fragments for 43 isolates of Dahongjun from southern China. For each isolate, the first 2–3 letters refer to their geographic locations (location labels are the same as in Figure 2) and the numerical digits refer to field isolation numbers. Numbers along branches are bootstrap values greater than 90% obtained from 1000 replicates. Tree length = 155, Consistency index = 0.718, Retention index = 0.885.

To examine whether the lineages and potentially sub-lineages identified above based on ITS sequences represented potentially reproductively isolated cryptic species, we sequenced three additional gene fragments for a selected subset of 43 isolates. These isolates included 35 strains in ITS Lineage 1 and four strains from each of Lineages 2 and 3 (Table 1 and Figures 2 and 3). At least two strains were included from each of the ITS sub-lineages mentioned above. In total, the sequence of 1753 additional nucleotides was obtained from each of the 43 isolates, including 902 bp from nucLSU rRNA (GenBank accession numbers GU352938-GU352980), 429 bp from mtSSU rRNA (GenBank accession numbers GU352981-GU353023), and 422 bp from RPB2 (GenBank accession numbers GU353024-GU353066), These sequences were aligned and analyzed. Our hypothesis was that if these three lineages and their sub-lineages represented reproductively isolated cryptic species, we should see consistent divergence among the four DNA fragments. In contrast, lack of consistent divergence among the genes would suggest that they belonged to the same reproductive group in nature [25], [26]. Below we describe briefly the patterns of sequence variation and the divergence of the three additional loci within and among the Dahongjun lineages and sub-lineages.

For the nucLSU rRNA DNA fragment, our analyses of the 902 aligned nucleotide sites identified a total of 13 variable sites among the 43 strains. These variable sites separated the 43 strains into 6 sequence types belonging to three phylogenetically well-supported groups (bootstrap values 84–99%; Figure S1). Specifically, these groups corresponded to the three lineages identified using ITS sequences (Figure 2 and Figure S1). Within Lineage 1, no sequence variation was found within either sub-lineage but difference at one nucleotide site was observed between the two sub-lineages. A single nucleotide difference also separated the two sub-lineages in Lineage 2. However, there were two nucleotides that differed between the two strains (DDG2-2 and JN24) within sub-lineage 1. Different from those in Lineages 1 and 2, no nucleotide variation was found within the nucLSU rRNA gene among the four sequenced strains in Lineage 3 (Figure S1).

For the mtSSU rRNA gene fragment, the analysis of the 429 nucleotides among the 43 isolates identified 2 variable sites and 3 genotypes. The three genotypes corresponded exactly to the three lineages identified based on ITS sequences. Different from the other three sequenced DNA fragments, there was no sequence variation among strains within any of the three lineages at the mtSSU rRNA locus (Figure S2).

For the RPB2 gene fragment, our analyses of the 422 nucleotides identified 22 variable sites and 26 genotypes among the 43 analyzed strains. The same three lineages revealed by ITS, nucLSU rRNA, and mtSSU rRNA gene sequences were also evident from the RPB2 sequences. There were two additional noteworthy features for this gene fragment. First, similar to the mtSSU rRNA gene fragment, there was no evidence for divergence between the pairs of sub-lineages within any of the three lineages (Figure S3). Second, while no sequence polymorphism was found within either Lineages 2 or 3, 13 nucleotide sites were found variable among the 35 strains within Lineage 1. These variable sites separated the 35 strains in Lineage 1 into 24 RPB2 sequence types. The two most frequent RPB2 genotypes here contained three strains each, 7 genotypes contained two strains each, and the remaining 15 genotypes contained one strain each (Figure S3).

Taken together, our genealogical analyses of the four DNA fragments suggest that the samples analyzed here represented three reproductively isolated phylogenetic species. Consistent with the observed phylogenetic pattern described above, the partition homogeneity test also failed to reject the hypothesis that the four gene genealogies were statistically congruent (p = 0.99). In addition, for each of the four datasets, we used the topology-dependent permutation test implemented in PAUP and constrained the tree topologies to fit into the three lineages. We found that none of the constrained MP trees were longer than the corresponding MP trees without any constraints and that the topological constraint for each of the gene fragment did not differ significantly from the topologies of other three gene fragments with the same constraints. These results are consistent with the reciprocal monophyly of the three lineages among the four sequenced gene fragments. A combined phylogeny based on concatenated sequences of all four DNA fragments (2434 bp) is presented in Figure 3.

### Haplotype inferences and evidence for recombination

In this study, while the primary objective was to examine the phylogenetic diversity of Dahongjun from southern China, the availability of multilocus sequence data for 33 strains of the Ailaoshan population (Lineage 1 in Figure 2) in central Yunnan offered us an opportunity to examine whether recombination played a role in generating the observed genetic diversity in this population. Typical tests for evidence of recombination within microbial populations examine the associations between alleles from the same locus (for diploid, dikaryotic, or heterokaryotic organisms) or among loci (for haploid to higher ploidy organisms). Statistically significant associations among alleles would be consistent with clonal reproduction while random associations among alleles would be consistent with sexual reproduction [26].

To examine the relationships among alleles within and between loci in the Ailaoshan population, we first inferred the alleles for each of the four DNA fragments for the 33 strains using the computer program PHASE 2.1 [27]. Of the four sequenced DNA fragments, two (nucLSU rRNA and mtSSU rRNA) were invariable among the 33 Ailaoshan strains and all these strains were homozygous through the entire sequenced fragment. As a result, these two gene fragments were not included for further analyses of population structure within the Ailaoshan population. The remaining two DNA fragments, ITS and RPB2, contained 6 and 13 variable sites respectively and they revealed 15 and 24 composite sequence types respectively within this population.

Within the ITS, four of the 6 variable nucleotide sites were heterozygous within at least one strain while the remaining two were homozygous within all strains but differed among the 33 strains. Of the 33 strains, eight were homozygous at all ITS nucleotide positions and the remaining 25 strains had 1 to 3 heterozygous sites each. At the RPB2 locus, 7 of the 13 variable nucleotide sites were heterozygous within at least one strain. Ten of the 33 strains were homozygous at all RPB2 nucleotide positions while the remaining 23 strains had 1 to 4 heterozygous sites each. When the genotype information from both the ITS and the RPB2 loci were combined, three of the 33 strains were homozygous at both loci, 15 strains were heterozygous at either the ITS or the RPB2 loci, and 15 strains were heterozygous at both loci. A total of 31 bi-locus (ITS and RPB2) genotypes were identified for the 33 isolates: two were represented by two specimens each while the remaining 29 genotypes were represented by one specimen each.

The PHASE analyses inferred 11 ITS haplotypes and 29 RPB2 haplotypes within the 33 strains. The relationships among the inferred haplotypes for the ITS and RPB2 DNA fragments for the 33 Ailaoshan strains are shown in Supplemental Figures S4 and S5 respectively. The haplotype (or allele) and genotype information were then analyzed for their associations with each other using the program Multilocus [28]. The results of our analyses were consistent with recombination in this natural population of Dahongjun. Specifically, the observed *I_A_* value was 0.02437, not significantly different from the distribution range of *I_A_* derived from 1000 randomized dataset (−0.08969 to 0.06463; p = 0.23). In addition, the two loci were phylogenetically incompatible within this population as demonstrated by the presence of all possible genotype combinations between pairs of haplotypes at the two loci. Specifically, two pairs of strains showed clear evidence of phylogenetic incompatibility: AL9-2-1 and AL9-5; and AL18-4 (or AL18-5) and AL7-1 (Figures S4 and S5).

## Discussion

In this study, we used DNA sequences to analyze the diversity and population structure within and among geographic samples of Dahongjun in southern China. Our phylogenetic analysis of the ITS genotypes revealed three distinct lineages among the commercially collected wild Dahongjun samples (Figure 2). The observed sequence divergences among the three lineages at the ITS locus were comparable to or greater than those between several of the closely related known *Russula* species (Figure 2). The distinctiveness of these three lineages was further supported by our analyses of DNA sequences from three other genes. The significant and consistent divergence among these lineages suggests that Dahongjun in southern China is a species complex and that each lineage represents a distinct phylogenetic species. Below we discuss the relevance of our results to the taxonomy, population genetics, and conservation biology of this mushroom in nature.

### Phylogenetics and taxonomy

Multiple gene genealogical analysis has become a common method to address issues of species recognition and to identify the patterns of genetic variation in natural microbial populations, including those of fungi [25], [26]. Indeed, the phylogenetic species concept based on gene genealogical congruence is now among the most common methods for identifying fungal species and many fungi previously thought to belong to a single species actually contain multiple phylogenetically distinct cryptic species [e.g. 29]–[35]. For example, the human fungal pathogens *Cryptococcus neoformans* [29], [30], *Candida* spp. [31], and *Histoplasma capsulatum* [32], the endemic gourmet mushroom from southwestern China *Thelephora ganbajun* [18], the mycorrhizal fungi *Paxillus involutus* [33] and *Cenococcum geophilum* [34], and the wood decay Boletales *Serpula himantioides* [35] have been identified as species complexes with each containing two or more cryptic species.

Our analyses identified that none of our samples had ITS sequences identical to *Russula vinosa*, a species previously thought to encompass Dahongjun from southern China (Figure 2). The distinctiveness of Dahongjun from other known *Russula* species had 100% bootstrap support, with large phylogenetic distances among them. These results thus indicate that none of the three Dahongjun lineages belonged to *R. vinosa* or likely any other *Russula* species described from Europe and North America, including the *Russula*-like sequences directly obtained so far in environmental DNA samples. In addition, the recently proposed *R. griseocarnosa* represented only one of the three lineages of Dahongjun (Lineage 2 in Figure 2). The detailed morphological, ecological, and further molecular characterization of Lineages 1 and 3 are in progress.

While the ITS sequences suggested that all three lineages contained additional sub-lineages, support from three other DNA fragments for the phylogenetic distinctiveness of the sub-lineages was weak or non-existent. Our result is consistent with the recent taxonomic description of *R. griseocarnosa* that included three sequences spanning both subgroups within Lineage 2 [20]. However, two of the three non-ITS DNA fragments analyzed here (nucLSU rRNA and mtSSU rRNA genes) are very conservative and not typically informative for separating closely related species or recently diverged populations. Therefore, we would like to point out that the current results do not exclude the possibility that some of the sub-lineages could be potentially reproductively isolated from each other. The analyses of additional DNA sequences from other genes in the Dahongjun genomes as well as the inclusion of more samples should help resolve the issue of additional reproductively isolated subgroups within each of the three lineages.

### Sequence diversity variation among gene fragments

Among the four DNA fragments analyzed here for the Ailaoshan population of Dahongjun, the two ribosomal rRNA genes (nucLSU rRNA and mtSSU rRNA) were much less variable than the ITS and RPB2 DNA fragments. The limited variation for the nucLSU rRNA and mtSSU rRNA sequences is likely due to their functional constraint at the nucleotide sequence level. Limited variation among strains within a species has also been observed in many other fungi at these two loci [e.g. 29], [ 31], [ 36]. Between ITS and RPB2, ITS was more variable and revealed more genotypes than RPB2 within Lineages 2 and 3. This might be due to the multi-copy nature of ITS sequences because paralogs containing substitutions would contribute to the number of sequence types observed here. However, RPB2 sequences revealed slightly more genotypes than ITS (24 vs 21) among the 35 strains within Lineage 1.

### Geographic patterns of sequence variation

Our phylogeographic analyses suggest that each of the three lineages identified here have relatively broad geographic distributions. Specifically, all three lineages contained specimens from both Yunnan and Guangxi provinces. However, within each of the three lineages (i.e. phylogenetic species), geography has likely played some role in the observed sequence variation. For example, the Yunnan and Guangxi samples were clustered separately in both Lineages 1 and 3 in the ITS phylogeny. Within Yunnan Province, Lineage 1 was found only in Ailaoshan in central Yunnan while Lineages 2 and 3 were found only in the south. These results are consistent with both ancient speciation among the three lineages and continuing divergence among geographic populations within individual lineages. Similar types of geographic patterns of DNA sequence variation have also been observed in many other fungal groups. For example, distinct alleles were observed in geographically separated populations of *Russula brevipes* [8]. Furthermore, in another gourmet mushroom *Th. ganbajun* from central and southern Yunnan, several potentially cryptic species were identified and the distributions of most cryptic species were geographically restricted, with the majority of the ITS sequence types found unique to one or a few close local populations [18]. These results are consistent with allopatric speciation.

While our results suggested that geographic separation contributed significantly to the observed sequence variation among and within individual lineages, the data also indicated evidence of dispersal and gene flow between some of the local and regional populations of Dahongjun. For example, all three lineages contained strains from both Yunnan and Guangxi, consistent with ancient and/or recent dispersal and gene flow. In addition, a few identical nucleotide sequences were found shared between strains from these two provinces at three of the four analyzed loci (Figures S1, S2, and S3). Though the sampled sites were separated by up to 1500 km from each other, the gene flow inferred above need not be long-distance dispersals but instead could be due to short-distance dispersals through as yet unidentified geographic populations of this group of organisms. Recent population genetic analyses suggested that short-distance spore dispersals are common while long-distance dispersals are relatively rare among ectomycorrhizal mushrooms (including species in the genus *Russula*) [37]–[42]. For example, empirical observations have shown that basidiospores can travel distances of up to 500 m by air currents in *Tricholoma matsutake* [41]. Similarly, spore dispersals up to 2090 m by squirrels were reported in several hypogeous and epigeous fungi [42]. Both short-distance and long-distance dispersals of fungi could be facilitated by wind, animals, humans, and human activities [36]. Indeed, infrequent gene flow over hundreds of kilometers has been inferred for several mushroom species from Yunnan, including the gourmet mushrooms *T. matsutake* and *T. ganbajun* [17]–[19], [43].

### Reproductive isolation, mode of reproduction, and resource management implications

Importantly, two geographic locations, DDG in southern Yunnan and CW in eastern Guangxi, contained strains belonging to two and three Dahongjun lineages respectively. The existence of strains from different lineages at the same geographic location but without allelic mixing is consistent with them being reproductively isolated from each other [34]. At present, the mechanism(s) for the potential reproductive isolation among these lineages remains unknown. Indeed, there is relatively little information about how these mushrooms reproduce in nature. Given the observed heterozygosity within strains at both the ITS and RPB2 loci and the random associations between their alleles in the Ailaoshan population, we speculate that the Ailaoshan population likely has a heterothallic life cycle and that sexual mating and recombination are important components of its reproduction. Among basidiomycete mushrooms, sexual mating and recombination may be accomplished between genetically compatible individuals either via inbreeding (between those closely related) or outcrossing (between genetically distantly related ones) [26]. Such recombination may involve either self-sterile haploid mycelia germinated from basidiospores and/or fertile diploid (or dikaryotic) mycelia [e.g. 44].

Our evidence for sexual recombination in the Ailaoshan population of Dahongjun has potentially significant implications for the management and conservation of this resource. At present, due to competition among mushroom hunters, an increasing proportion of the Dahongjun mushrooms are picked at the immature stage, before the opening of mushroom cap and the release of their basidiospores. As a result, mushrooms that could have contributed to the future reproduction of these populations in nature were increasingly eliminated from the gene pool by mushroom hunters. If this trend continues, the genetic diversity of many of these populations may decrease rapidly, leading to potential population extinctions. Indeed, many local geographic populations of the gourmet mushroom *T. matsutake* around Kunming, the capital city of Yunnan Province, have disappeared over the last 20 years, likely due to habitat loss, uncontrolled over-harvesting of immature fruiting bodies and/or to physical damages to the underground mycorrhizae while searching for the mushrooms [19], [43].

In conclusion, our study suggests that the commercially collected Dahongjun is a species complex, composed of at least three distinct lineages. More extensive sampling and the analyses using other gene sequences may reveal additional distinct lineages as well as novel distribution patterns within this species complex in southern China. The geographic distribution patterns indicate that reproductive isolation have likely occurred among these three lineages. Within at least one Dahongjun population in central Yunnan, the Ailaoshan population, we observed evidence of recombination, likely involving basidiospores. While our data indicated evidence for ancient and/or recent dispersal and gene flow among these geographic populations, such events are relatively limited. Our results suggest that care should be taken to ensure that the genetic diversity is maintained within these local populations to ensure the survival and continued commercial harvesting of this ecologically and economically important mushroom.

## Materials and Methods

### Sampling

Fruiting body samples of Dahongjun were collected from tropical and subtropical evergreen forests dominated by *Castanopsis* spp. and *Lithocarpus* spp. in Yunnan and Guangxi Provinces during the summers of 2006 and 2007. Because of their commercial value and non-cultivable nature, there has been significant competition among local residents within each area for the wild mushroom. Most local mushroom pickers guard their own secret mushroom fruiting spots in the forests and are unwilling to share the information with others. In addition, the most desirable Dahongjun fruiting bodies are the immature ones (those with their caps closed), picked while still buried underground and not visible by the naked eye. As a result, it was extremely difficult for those who are not familiar with the local terrains and fruiting spots to find the fruiting bodies. The mushrooms analyzed here were collected with the help of one picker at each location who, through various means, were assured the confidentiality of their fruiting spots. All the fruiting bodies collected during two-day forays at each location were included. These specimens were identified based on their morphological features as determined by the local mushroom hunters and tradesmen. A total of 122 fruiting bodies were collected from 4 areas in Yunnan Province and one area in Guangxi Province. These regions were chosen for this study because of Dahongjun trading activities noted by mushroom traders we encountered. The areas are separated by about 1500 km from east to west and about 500 km from north to south. The geographical locations of the sampled sites are shown in Figure 1. The geographic coordinates and the sample size from each site are presented in Table 1.

### DNA extraction and sequencing

Genomic DNA was extracted from dried fruiting bodies, using a CTAB miniprep method [45]. The extracted DNA was suspended in 50 µl TE buffer, and then diluted 1∶20 for use in PCR reactions. The ITS sequence was obtained for each of the 122 mushrooms. The primers ITS1 (5′-TCCGTAGGTGAACCTGCGG-3′) and ITS4 (5′-TCCTCCGCTTATTGATAGC-3′) were used to amplify the ITS regions of the ribosomal RNA gene cluster that included ITS1, 5.8S, and ITS2 [24]. Each PCR reaction contained 10 µl reaction volume: 5 µl GoTaq**®** Green Master Mix 2× (Promega), 1.5 µl diluted genomic DNA, 1 µM of each primer, and 1.5 µl ddH2O. The PCR was conducted with the following program: 4 min at 94°C, followed by 35 cycles of 30 s at 94°C, 30 s at 50°C, and 90 s at 72°C, and a final extension step at 72°C for 10 min. PCR products were then cleaned and sequenced using cycle sequencing with BigDye®Terminator v3.1 (Applied Biosystems) following the manufacturer's protocol. Sequencing was done in both forward and reverse directions for each sample using the ITS1 and ITS4 primers. The DNA sequencing was run at the MoBix Laboratory in the Department of Biology at McMaster University, using an ABI3100 automated DNA sequencer.

Portions of three other genes were analyzed for a sub-set of 43 isolates: the nuclear large subunit of the ribosomal RNA gene (nucLSU rRNA), the mitochondrial small subunit of the ribosomal RNA gene (mtSSU rRNA), and the second largest subunit of the nuclear RNA polymerase enzyme II (RPB2). The primers for amplifying each of these three gene fragments are as follows: LR0R (5′ ACCCGCTGAACTTAAGC 3′) and LR5 (5′ TCCTGAGGGAAACTTCG 3′) for nucLSU rRNA gene; MS1 (5′ AGCAGTGAGGAATATTGGTC 3′) and MS2 (5′ CTGACGTTGAAGGACGAAGG 3′) for the mtSSU rRNA gene; and bRPB2-6F (5′ TGGGGYATGGTNTGYCCYGC 3′) and bRPB2-7R (5′ GAYTGRTTRTGRTCRGGGAAVGG 3′) for the RPB2 gene. The primer sequences were obtained from the Assembly of the Fungal Tree of Life (AFTOL) website (http://aftol.org/primers.php).

### Sequence alignments and genotype identification

DNA sequence data obtained from the forward and reverse directions were assembled for each strain using the SeqMan sequence analysis software (DNASTAR, Inc.). All the heterozygous sites were coded using the following universal ambiguity codes: T/C = Y, A/G = R, A/C = M, G/T = K. Sequences were then aligned using Clustal_X version 2 [23], and the alignment was manually adjusted as needed. The aligned sequences were then input into PAUP*4.0b10 [24] to identify unique sequence types for each of the four sequenced DNA fragments. All aligned nucleotide sites, including the insertions/deletions, were used to identify sequence polymorphisms at each of the four loci.

### Phylogenetic analyses

To compare our samples with those in the databases, our ITS genotypes were used as queries to retrieve closely related sequences (≥90% sequence identities) with comparable lengths from GenBank. Whenever possible, multiple representative ITS sequences from the GenBank for each of the closely related *Russula* species were also included to compare potential intra-specific variation of the Dahongjun phylogenetic groups to those within known *Russula* species. ITS sequences of *Russula griseocarnosa*, which was recently described as a new species representing Dahongjun from southern Yunnan [20], were also included as reference sequences in our phylogenetic analysis. Our own sequences and all the retrieved sequences from GenBank were then aligned using Clustal_X version 2 [24]. The aligned sequences were visually inspected, adjusted and imported into PAUP*4.0b10 [24]. Maximum parsimony and maximum likelihood analyses were carried out. Topological support was estimated by bootstrap resampling of 1000 randomized datasets.

To examine whether the divergent lineages revealed by the ITS sequences represented potentially reproductively isolated cryptic species, we tested for congruence between genealogies from different loci using the partition homogeneity test and the topology-dependent permutation test. Consistent divergence among groups of isolates across different genes would indicate the existence of cryptic species, with each clade constituting a phylogenetic species [25], [46]. These analyses were conducted using PAUP software [24].

### Haplotype inference and test for the mode of reproduction

Fruiting bodies of mushrooms such as Dahongjun can release a large number of meiotic spores. If these sexual spores contributed to the reproduction of the populations in nature, we should be able to detect signatures of random associations among alleles at the same or different loci. To identify such signatures, we first obtained allelic information from each of the four loci for the 43 strains. Among the four sequenced DNA fragments, two (nucLSU rRNA and mtSSU rRNA) were completely homozygous within each strain (see Results above) while ITS and RPB2 contained heterozygous sites. The putative haplotype information for ITS and RPB2 was inferred using the PHASE 2.1 program [27]. Briefly, all variable nucleotide sites for ITS and RPB2 were selected and compiled into a required format for input into PHASE and the possible haplotypes and their frequencies within the samples were estimated using Bayesian analysis. Our samples were treated as dikaryons (diploid genotypes) analyzed under the biallelic (SNP) loci option. If a DNA sequence contained heterozygous sites, the resulting haplotypes would have only one base at each site. For sequences with no heterozygous positions, they were considered to contain a single homozygous haplotype. Each unique haplotype from this output was assigned a specific allele designation.

To examine the association among alleles and to test for the mode of reproduction, we chose the Ailaoshan population because of the availability of genotype information from a relatively large number of strains. Two common tests for assessing allelic associations were conducted here: the index of association (*I_A_*) and phylogenetic incompatibility tests [26]. Both the *I_A_* and the phylogenetic incompatibility tests were conducted using the program Multilocus version 1.0b [28]. The basic principles and underlying statistics for these two tests can be found in the program manual (28).

As was pointed out in previous sections of this manuscript, the ITS regions are repeated multiple times within the ribosomal gene cluster on each chromosome. Therefore, the heterozygous sites might not represent nucleotides from the two different chromosomes but instead could be due to variation among paralogs within a single chromosome. Consequently, caution should be taken when analyzing population structure using haplotype information from ITS sequences.

## Supporting Information

Table S1 Polymorphic nucleotide sites among the 63 ITS genotypes of Dahongjun. The corresponding positions for the variable nucleotides are shown. “-” represents indels.
(0.06 MB DOC)

Table S2 Within-strain sequence polymorphism at the ITS region for samples of Dahongjun analyzed in this study. AL: Ailaoshan; DG: Dadugang; ML: Mengla; JN: Jinuo; CW: Cangwu.
(0.07 MB DOC)

Figure S1 Maximum parsimony tree based on the nuclear large subunit ribosomal RNA (nucLSU rRNA) sequences from 43 representative isolates of *Russula* spp. collected from 5 study sites in 3 regions in southern China. Each strain is represented by its geographic affiliation (AL: Ailaoshan, central Yunnan; DDG: Dadugang, southern Yunnan; JN: Jinuo, southern Yunnan; ML: Mengla, southern Yunnan; and CW: Cangwu, eastern Guangxi) and one or more numbers representing our collection identification. Bootstrap support values (1000 replicates) are given above branches. Tree length = 15, Consistency index = 1, Retention index = 1.
(0.16 MB TIF)

Figure S2 Maximum parsimony tree based on the mitochondrial small subunit ribosomal RNA (mtSSU rRNA) sequences from 43 representative isolates of *Russula* spp. collected from 5 study sites in 3 regions in southern China. Strain labels are identical to those in Figure S1. Bootstrap support values (1000 replicates) are given above branches. Tree length = 2, Consistency index = 1, Retention index = 1.
(0.16 MB TIF)

Figure S3 Maximum parsimony tree based on the nuclear RNA polymerase II large subunit (RPB2) from 43 representative isolates of *Russula* spp. collected from 5 study sites in 3 regions in southern China. Strain labels are identical to those in Figure S1. Bootstrap support values (1000 replicates) are given above branches. Tree length = 49, Consistency index = 0.673, Retention index = 0.853.
(0.17 MB TIF)

Figure S4 Maximum parsimony tree based on the inferred ITS haplotype sequences of 33 strains from Ailaoshan. Each ITS haplotype is represented by its geographic affiliation (AL: Ailaoshan, central Yunnan), one or more numbers representing our field collection identification, followed by a or b that represent the two alleles within an individual specimen. Tree length = 10, Consistency index = 0.6, Retention index = 0.955.
(0.20 MB TIF)

Figure S5 Maximum parsimony tree based on the inferred RPB2 haplotype sequences of 33 strains from Ailaoshan. Each RPB2 haplotype is represented by its geographic affiliation (AL: Ailaoshan, central Yunnan), one or more numbers representing our field collection identification, followed by a or b that represent the two alleles within an individual specimen. Tree length = 35, Consistency index = 0.486, Retention index = 0.860.
(0.21 MB TIF)

## References

[1] Brundrett M (2004). Diversity and classification of mycorrhizal associations.. Biological Review - Cambridge Philosophical Society.

[2] Kirk PM, Cannon PF, Minter DW, Stalpers JA (2008). Dictionary of the Fungi, 10th edition..

[3] Li M, Xu J (2009). Molecular ecology of ectomycorrhizal fungi: molecular markers, genets and ecological importance.. Acta Botannica Yunnanica.

[4] Horton TR, Bruns TD (2001). The molecular revolution in ectomycorrhizal ecology: peeking into the black-box.. Molecular Ecology.

[5] Yang ZL, Piepenbring M, Agerer R, Piepenbring M, Blanz (2004). Wild edible fungi in the Yunnan Province, southwestern China.. Frontiers in Basidiomycote Mycology. IHW-Verlag.

[6] Romagnesi H (1967). Les Russules d'Europe et d'Afrique du Nord..

[7] Singer R (1986). The Agaricales in Modern Taxonomy. 4th edition..

[8] Bergemann SE, Miller SL (2002). Size distribution, and persistence of genets in local populations of the late-stage ectomycorrhizal basidiomycete, *Russula brevipes*.. New Phytologist.

[9] Bergemann SE, Miller SL, Garbelotto M (2005a). Microsatellite loci from *Russula brevipes*, a common ectomycorrhizal associate of several tree species in North America.. Molecular Ecology Notes.

[10] Bergemann SE, Douhan GW, Garbelotto M, Miller SL (2005b). No evidence of population structure across three isolated subpopulations of *Russula brevipes* in oak/pine woodland.. New Phytologist.

[11] Buyck B, Mitchell D, Parrent J (2006). *Russula parvovirescens* sp. nov., a common but ignored species in the eastern United States.. Mycologia.

[12] Miller SL, Buyck B (2002). Molecular phylogeny of genus *Russula* in Europe with a comparison of modern infrageneric classification.. Mycological Research.

[13] Richardson MJ (1970). Studies of *Russula emetica* and other agarics in a Scots pine plantation.. Transactions of the British Mycological Society.

[14] Myers N, Mittermeier RA, Mittermerier CG (2000). Biodiversity hotspots for conservation priorities.. Nature.

[15] Yang ZL (2002). On wild mushroom resources and their utilization in Yunnan Province, Southwest China.. Journal of Natural Resources.

[16] Lian B, Zang JP, Hou WG, Yuan S, Smith DL (2008). PCR-based sensitive detection of the edible fungus *Boletus edulis* from rDNA ITS sequences.. Journal of Biotechnology.

[17] Sha T, Zhang H, Ding H, Li Z, Cheng L (2007). Genetic diversity of *Tricholoma matsutake* in Yunnan Province.. Chinese Science Bulletin.

[18] Sha T, Xu J, Palanichamy MG, Zhang HB, Li T (2008). Genetic diversity of the endemic gourmet mushroom *Thelephora ganbajun* from southwestern China.. Microbiology.

[19] Xu J, Sha T, Li YC, Zhao ZW, Yang ZL (2008). Recombination and genetic differentiation among natural populations of the ectomycorrhizal mushroom *Tricholoma matsutake* from southwestern China.. Molecular Ecology.

[20] Wang XH, Yang ZL, Li YC, Knudsen H, Liu PG (2009). *Russula griseocarnosa* sp. nov. (Russulaceae, Russulales), a commercially important edible mushroom in tropical China: mycorrhiza, phylogenetic position, and taxonomy.. Nova Hedwigia.

[21] Legon NW, Henrici A, Roberts PJ, Walting R (2005). Checklist of the British and Irish Basidiomycota.. Royal Botanic Gardens, Kew.

[22] Tedersoo L, Kõljalg U, Hallenberg N, Larsson KH (2003). Fine scale distribution of ectomycorrhizal fungi and roots across substrate layers including coarse woody debris in a mixed forest.. New Phytologist.

[23] Thompson JD, Gibson TJ, Plewniak F, Jeanmougin F, Higgins DG (1997). The CLUSTAL_X–Windows interface: flexible strategies for multiple sequence alignment aided by quality analysis tools.. Nucleic Acids Research.

[24] Swofford DL (2002). PAUP*: Phylogenetic analysis using parsimony (and other methods), version 4..

[25] Taylor JW, Jacobson DJ, Kroken S, Kasuga T, Geiser DM (2000). Phylogenetic species recognition and species concepts in fungi.. Fungal Genetics and Biology.

[26] Xu J, Xu J (2005). Fundamentals of fungal molecular population genetic analyses..

[27] Stephens M, Donnelly P (2003). A comparison of Bayesian methods for haplotype reconstruction.. American Journal of Human Genetics.

[28] Agapow PM, Burt A (2001). Indices of multilocus linkage disequilibrium.. Molecular Ecology Notes.

[29] Xu J, Vilgalys R, Mitchell TD (2000). Multiple gene genealogies reveal recent dispersion and hybridization in the human pathogenic fungus *Cryptococcus neoformans*.. Molecular Ecology.

[30] Bovers M, Hagen F, Kuramae EE, Boekhout T (2008). Six monophyletic lineages identified within *Cryptococcus neoformans* and *Cryptococcus gattii* by multi-locus sequence typing. Fungal Genetics and Biology..

[31] Pujol C, Dodgson A, Soll DR (2005). Population genetics of ascomycetes pathogenic to humans and animals..

[32] Kasuga T, White TJ, Koenig GL, McEwen JG, Restrepo A (2003). Phylogeography of the fungal pathogen *Histoplasma capsulatum*.. Molecular Ecology.

[33] Hedh J, Samson P, Erland S, Tunlid A (2008). Multiple gene genealogies and species recognition in the ectomycorrhizal fungus *Paxillus involutus*.. Mycological research.

[34] Douhan GW, Rizzo DM (2005). Phylogenetic divergence in a local population of the ectomycorrhizal fungus *Cenococcum geophilum*.. New Phytologist.

[35] Kauserud H, Stensrud Ø, DeCock C, Shalchian-Tabrizi K, Schumacher T (2006). Multiple gene genealogies and AFLPs suggest cryptic speciation and long-distance dispersal in the basidiomycete *Serpula himantioides* (Boletales).. Molecular Ecology.

[36] Xu J, Cheng M, Tan Q, Pan Y, Xu J (2005). Molecular population genetics of basidiomycete fungi..

[37] Lamb RJ (1979). Factors responsible for the distribution of mycorrhizal fungi of *Pinus* in eastern Australia.. Australian Journal of Forest Research.

[38] Allen M, Phipps LE (1984). Comparative microlimates of some mycorrhizal fungi: requirements for long-range dispersal of sproes.. Mycological Society of America Newsletter.

[39] Redecker D, Szaro TM, Bowman RJ, Bruns TD (2001). Small genets of *Lactarius xanthogalactus*, *Russula cremoricolor* and *Amanita francheti* in late-stage ectomycorrhizal successions.. Molecular Ecology.

[40] Liang Y, Guo LD, Ma KP (2004). Genetic structure of a population of the ectomycorrhizal fungus *Russula vinosa* in subtropical woodlands in southwest China.. Mycorrhiza.

[41] Lian CL, Narimatsu M, Nara K, Hogetsu T (2006). *Tricholoma matsutake* in a natural *Pinus densiflora* forest: correspondence between above- and below-ground genets, association with multiple host trees and alteration of existing ectomycorrhizal communities.. New Phytologist.

[42] Bertolino S, Vizzini A, Wauters LA, Tosi G (2004). Consumption of hypogeous and epigeous fungi by the red squirrel (*Sciurus vulgaris*) in sub-alpine conifer forests.. Forest Ecology and Management.

[43] Xu J, Guo H, Yang ZL (2007). Single nucleotide polymorphisms in the ectomycorrhizal mushroom *Tricholoma matsutake*.. Microbiology.

[44] Xu J, Horgen PA, Anderson JB (1996). Somatic recombination in the cultivated mushroom *Agaricus bisporus*.. Mycological Research.

[45] Xu J, Yoell HJ, Anderson JB (1994). An efficient protocol for isolating DNA from higher fungi.. Trends Genet.

[46] Farris JS, Källersjö M, Kluge AG, Bult C (1994). Testing significance of incongruence.. Cladistics.

