# Supplementary material for: Genetic Diversity of Dahongjun, the Commercially Important “Big Red Mushroom” from Southern China
Source: PLoS One. 2010 May 18;5(5):e10684. doi: 10.1371/journal.pone.0010684 (PMC2872671; doi:10.1371/journal.pone.0010684)
Supplement: Table S1 — Polymorphic nucleotide sites among the 63 ITS genotypes of Dahongjun. The corresponding positions for the variable nucleotides are shown. “-” represents indels. (0.06 MB DOC) [file pone.0010684.s001.doc]

Supplemental Table S1. Polymorphic nucleotide sites among the 63 ITS genotypes of Dahongjun. The corresponding positions for the variable nucleotides are shown. “-” represents indels.

Nucleotide positions

0000000000001111111111111111111113333333333333444444444444444444444444444444455555

3555566677781233333456666778888894778888999999000000011111111222344566666677934569

Genotype 4025824617939601234552349361378930453456024567035678901234567026046902678905358594

Geno_56 ACTTGYTCTAATATTCGCATTTCGCTAT-CCTCACCC-----T-----------------GGATTCCCACTTGCGTTTG-GC

Geno_57 ACTTGTTCTAATATTCGCATTTCGCTAT-CCTCACCC-----T-----------------GGATTCCCACTTGCGTTTG-GC

Geno_55 ACTTGYTCTAATATTTGCATTTCGCTAT-CCTCACCC-----T-----------------GGATTCCCACTTGCGTTTG-GC

Geno_58 ACT-TCTCCAATACTCGCATTTCGCTAT-CCTCACCC-----T-----------------GGATTCCCACTTGCGTTTG-GC

Geno_39 ACTTGTTCTAATATTTGCATT-CGTTGT-ACTCCTTY-Y-CCTCCTTT-TTGGTCAGGGAAAGGACT-CACTGCGCTTG-GC

Geno_42 ACTTGTTCTAATATTTGCATT-CGTTGT-ACTCCTTT-YTCCTCCTTT-TTGGTCAGGGAAAGGACT-CACTGCGCTTG-GC

Geno_46 ACTTGTTCTAATATTTGCATT-CGTTGT-ACTCCTTT-CTCCTCCTTT-TTGGTCAGGGAAAGGACTTCACTGCGCTTG-GC

Geno_38 ACTTGTTCTAATATTTGCATT-CGTTGT-ACTCCTT--CTCCTCCTTT-TTGGTCAGGGAAAGGACT-CACTGCGCTTG-GC

Geno_44 ACTTGTTCTAATATTTGCATT-CGTTGT-ACTCCTTTTTCCCTCCTTT-TTGGTCAGGGAAAGGACT-CACTGCGCTTG-GC

Geno_45 ACTTGTTCTAATATTTGCATT-CGTTGT-ACTCCTTT--CCCTCCTTT-TTGGTCAGGGAAAGGACT-CACTGCGCTTG-GC

Geno_43 ACTTGTTCTAATATTTGCATT-CGTTGT-ACTCCTTT--TCCTCCTTT-TTGGTCAGGGAAAGGACT-CACTGCGCTTG-GC

Geno_48 ACTTGTTCTAATATTTGCATT-CGTTGT-ACTCCTTT-CYCCTCCTTT-TTGGTCAGGGAAAGGACT-CACTGCGCTTG-GC

Geno_47 ACTTGTTCTAATATTTGCATT-CGTTGT-ACTCCTTT-CYC-TCCTTT-TTGGTCAGGGAAAGGACT-CACTGCGCTTG-GC

Geno_49 ACTTGYTCTAATATTTGCATT-CGTTGT-ACTCCTTT-CYC-TCCTTT-TTGGTCAGGGAAAGGACT-CACTGCGCTTG-GC

Geno_41 ACTTGYTCTAATATTTGCATT-CGTTGT-ACTCCTTT--TCCTCCTTT-TTGGTCAGGGAAAGGACT-CACTGCGCTTG-GC

Geno_40 ACTTGYTCTAATATTTGCATT-CGTTGT-ACTCCTTY--YCCTCCTTT-TTGGTCAGGGAAAGGACT-CACTGCGCTTG-GC

Geno_50 ACTTGYTCTAATATTTGCATT-CGTTGT-ACTCCTTT--CCCTCCTTT-TTGGTCAGGGAAAGGACT-CACTGCGCTTG-GC

Geno_51 ACTTGTTCTAATATTTGCATT-CGTTGT-ACTCCTTT--CCCTCCTTT-TTGGTCAGGGAAAGGACT-CACTGCGCTTG-GC

Geno_53 ACTTGCTCTAATATTTGCATT-CGTTGT-ACTCCTTT--CCCTCCTTT-TTGGTCAGGGAAAGGACT-CACTGCGCTTG-GC

Geno_52 ACTTGCTCTAATATTTGCATT-CGTTGT-ACYCCTTT--CCCTCCTTT-TTGGTCAGGGAARGGACT-CACTGCGCTTG-GC

Geno_54 ACTTGCTCTAATACTTGCACT-CGTTAT-ACTACATT-CCCCTCCTTT-TTGGTCAGGGAAAGGACT-CACTGCGCTTG-GC

Geno_33 GCT-CCTCCGTAGCTTGCATT-CACTATTCTTCCTCC-----TCCTTCATCGGTCAGGGAAAGGACTTCACTGCGCTCG-GC

Geno_32 GCT-CCTCCRTAGCTTGCATT-CACTATTCTTCCTCC-----TCCTTCATCGGTCAGGGAAAGGACTTCACTGCGCTCG-GC

Geno_26 GCT-CCTCCGTAGCTTGCATT-CACTATTCTTCCTCC-----TCCTTCATCGGTCAGGGAAAGGACTTCACTGCGCTCG-GM

Geno_25 GCT-CCTCCGTAGCTTGCATT-CACTATTCTTCCTCC-----TCCTTCATCGGTCAGGGAAAGGACTTCACTGCGCTCG-GC

Geno_34 GCT-CCTCCRTAGCTTGCATT-CACTAYTCTTCCTCC-----TCCTTCATCGGTCAGGGAAAGGACTTCACTGCGCTCG-GC

Geno_30 GCT-CCTCCGTAGCTTGCATC-CACTATTCTTCCTCC-----TCCTTCATCGGTCAGGGAAAGGACTTCACTGCGCTCG-GC

Geno_31 GCT-CCTCCGTAGCTTGCATC-CACTATTCTTCCTCC-----TCCTTCATCGGTCAGGGAAAGGACTTCACTGCGCTCG-GC

Geno_27 GCT-CCTCCGTAGCTTGCATY-CACTATTCTTCCTCC-----TCCTTCATCGGTCAGGGAAAGGACTTCACTGCGCTCG-GC

Geno_28 GCT-CCTCCGTAGCTTGCATY-CACTATTCTTCCTCC-----TCCTTCATCGGTCAGGGAAAGGACTTCACTGCGCTCG-GC

Geno_29 GCT-CCTCCATAGCTTGCATY-CACTATTCTTCCTCC-----TCCTTCATCGGTCAGGGAAAGGACTTCACTGCGCTCG-GM

Geno_23 GCT-CCTCCATAGCTTGCGTY-CACTATTCTTCCTCC-----YCCTTCATCGGTCAGGGAAAGGACTTCACTGCTCTCG-GC

Geno_35 GCT-CCTCCATAGCTTGCATY-CACTATTCTTCCTCC-----TCCTTCATCGGTCAGGGAAAGGACTTCACTGCGCTCG-GC

Geno_10 GCT-CCTCCRTAGCTTGCRTY-CACTATTCTTCCTCC-----TCCTTCATCGGTCAGGGAAAGGACTTCACTGCKCTCG-GC

Geno_11 GCT-CCYCCRTAGCTTGCRTY-CACTATTCTTCCTCC-----YCCTTCATCGGTCAGGGAAAGGACTTCACTGCKCTCG-GC

Geno_12 GCT-CCTCCRTAGCTTGCRTY-CACTATTCTTCCTCC-----TCCTTCATCGGTCAGGGAAAGGACTTCACTGCKCTCG-GC

Geno_13 GCT-CCTCCRTAGCTTGCRTY-CACTATTCTTCCTCC-----TCCTTCATCGGTCAGGGAAAGGACTTCACTGCKCTCG-GC

Geno_14 GCT-CCTCCRTAGCTTGCRTY-CACTATTCTTCCTCC-----TCCTTCATCGGTCAGGGAAAGGACTTCACTGCGCTCG-GC

Geno_17 GCT-CCTCCRTAGCTTGCATT-CACTATTCTTCCTCC-----TCCTTCATCGGTCAGGGAAAGGACTTCACTGCKCTCG-GC

Geno_15 GCT-CCTCCRTAGCTTGCGTT-CACTATTCTTCCTCC-----TCCTTCATCGGTCAGGGAAAGGACTTCACTGCKCTCG-GC

Geno_16 GCT-CCTCCRTAGCTTGCRTT-CACTATTCTTCCTCC-----TCCTTCATCGGTCAGGGAAAGGACTTCACTGCKCTCG-GM

Geno_18 GCT-CCTCCGTAGCTTGCGTT-CACTATTCTTCCTCC-----TCCTTCATCGGTCAGGGAAAGGACTTCACTGCGCTCG-GC

Geno_19 GCT-CCTCCGTAGCTTGCRTT-CACTATTCTTCCTCC-----TCCTTCATCGGTCAGGGAAAGGACTTCACTGCKCTCG-GC

Geno_08 GCT-CCTCCATAGCTTGCRTT-CACTATTCTTCCTCC-----TCCTTCATCGGTCAGGGAAAGGACTTCACTGCKCTCG-RC

Geno_09 GCT-CCTCCATAGCTTGCRTT-CACTATTCTTCCTCC-----TCCTTCATCGGTCAGGGAAAGGACTTCACTGCKCTCG-RC

Nucleotide positions

0000000000001111111111111111111113333333333333444444444444444444444444444444455555

3555566677781233333456666778888894778888999999000000011111111222344566666677934569

Genotypes4025824617939601234552349361378930453456024567035678901234567026046902678905358594

Geno_24 GCT-CCTCCATAGCTTGCRTT-CACTATTCTTCCTCC-----TCCTTCATCGGTCAGGGAAAGGACTTCACTGCTCTCG-GC

Geno_07 GCT-CCTCCATAGCTTGCRTT-CACTATTCTTCCTCC-----TCCTTCATCGGTCAGGGAAAGGACTTCACTGCKCTCG-GC

Geno_02 GCT-CCTCCATAGCTTGCRTT-CACTATTCTTCCTCC-----TCCTTCATCGGTCAGGGAAAGGACTTCACTGCKCTCG-GC

Geno_03 GCT-CCTCCRTAGCTTGCRTT-CACTATTCTTCCTCC-----TCCTTCATCGGTCAGGGAAAGGACTTCACTGCKCTCG-GC

Geno_04 GCT-CCTCCATAGCTTGCRTT-CACTAYTCTTCCTCC-----TCCTTCATCGGTCAGGGAAAGGACTTCACTGCKCTCG-GC

Geno_05 GCT-CCTCCRTAGCTTGCRTT-CACTATTCTTCCTCC-----TCCTTCATCGGTCAGGGAAAGGACTTCACTGCKCTCR-RC

Geno_06 GCT-CCTCCATAGCTTGCRTT-CACTATTCTTCCTCC-----TCCTTCATCGGTCAGGGAAAGGACTTCACTGCKCTCR-RC

Geno_01 GCT-CCTCCATAGCTTGCRTT-CACTATTCTTCCTCC-----TCCTTCATCGGTCAGGGAAAGGACTTCACTGCGCTCG-GC

Geno_22 GCT-CCTCCATAGCTTGCGTT-CACTATTCTTCCTCC-----TCCTTCATCGGTCAGGGAAAGGACTTCACTGCTCTCG-RC

Geno_21 GCT-CCTCCATAGCTTGCGTT-CACYATTCTTCCTCC-----TCCTTCATCGGTCAGGGAAAGGACTTCACTGCTCTCR-RC

Geno_20 GCT-CCTCCATAGCTTGCGTT-CACTATTCTTCCTCC-----TCCTTCATCGGTCAGGGAAAGGACTTCACTGCTCTCG-GC

Geno_37 GCT-CCTCCATAGCTTGCGTT-CACTATTCTTCCTCC-----TCCTTCATCGGTCAGGGAAAGGACTTCACTGCGCTCG-GC

Geno_36 GCT-CCTCCATAGCTTGCATT-CACTATTCTTCCTCC-----TCCTTCATCGGTCAGGGAAAGGACTTCACTGCGCTCG-RC

Geno_61 GCT-CCTCCATAGCGCAAATT-CGTTATTCTTCCTCC-----YCCTTCATCGGTCAGGGAAAGGATTTCACCGCGCCTGTGC

Geno_59 GCT-CCTCCATAGCGCAAATT-CGTTATTCTTCCTCC-----CCCTTCATCGGTCAGGGAAAGGATTTCACCGCGCCTGTGC

Geno_60 GCT-CCTCCATAGCGCAAATT-CGTTATTCTTCCTCC-----TCCTTCATCGGTCAGGGAAAGGATTTCACCGCGCCTGTGC

Geno_62 GCT-CCTCCATAGCGCAAATT-CGTTATTCTTCCTCC-----CCCTTCATCGGTCAGGGAAAGGAYYTCACYGCGCYTGTGC

Geno_63 GCT-CCTCCATAGCGCAAATT-CGTTATTCTTCCTCC-----YCCTTCATCGGTCAGGGAAAGGAYYTCACYGCGCYTGTGC
