# Supplementary material for: Genetic Diversity of Dahongjun, the Commercially Important “Big Red Mushroom” from Southern China
Source: PLoS One. 2010 May 18;5(5):e10684. doi: 10.1371/journal.pone.0010684 (PMC2872671; doi:10.1371/journal.pone.0010684)
Supplement: Table S2 — Within-strain sequence polymorphism at the ITS region for samples of Dahongjun analyzed in this study. AL: Ailaoshan; DG: Dadugang; ML: Mengla; JN: Jinuo; CW: Cangwu. (0.07 MB DOC) [file pone.0010684.s002.doc]

Supplemental Table S2. Within-strain sequence polymorphism at the ITS region for samples of Dahongjun analyzed in this study. AL: Ailaoshan; DG: Dadugang; ML: Mengla; JN: Jinuo; CW: Cangwu.

| Isolate | Group | No. of heterozygous  sites within ITS regions | Geographic  Origin |
| --- | --- | --- | --- |
| DahongjunGeno01 | Lineage 1 | 1 | AL |
| DahongjunGeno02 | Lineage 1 | 2 | AL |
| DahongjunGeno03 | Lineage 1 | 3 | AL |
| DahongjunGeno04 | Lineage 1 | 3 | AL |
| DahongjunGeno05 | Lineage 1 | 5 | AL |
| DahongjunGeno06 | Lineage 1 | 4 | AL |
| DahongjunGeno07 | Lineage 1 | 2 | AL |
| DahongjunGeno08 | Lineage 1 | 3 | AL |
| DahongjunGeno09 | Lineage 1 | 5 | AL |
| DahongjunGeno10 | Lineage 1 | 4 | AL |
| DahongjunGeno11 | Lineage 1 | 6 | AL |
| DahongjunGeno12 | Lineage 1 | 4 | AL |
| DahongjunGeno13 | Lineage 1 | 4 | AL |
| DahongjunGeno14 | Lineage 1 | 3 | AL |
| DahongjunGeno15 | Lineage 1 | 2 | AL |
| DahongjunGeno16 | Lineage 1 | 3 | AL |
| DahongjunGeno17 | Lineage 1 | 2 | AL |
| DahongjunGeno19 | Lineage 1 | 1 | AL |
| DahongjunGeno21 | Lineage 1 | 3 | AL |
| DahongjunGeno22 | Lineage 1 | 1 | AL |
| DahongjunGeno23 | Lineage 1 | 2 | AL |
| DahongjunGeno24 | Lineage 1 | 2 | AL |
| DahongjunGeno27 | Lineage 1 | 1 | AL |
| DahongjunGeno28 | Lineage 1 | 1 | AL |
| DahongjunGeno29 | Lineage 1 | 1 | AL |
| DahongjunGeno32 | Lineage 1 | 1 | AL |
| DahongjunGeno34 | Lineage 1 | 1 | AL |
| DahongjunGeno35 | Lineage 1 | 1 | AL |
| DahongjunGeno36 | Lineage 1 | 1 | AL |
| DahongjunGeno61 | Lineage 1 | 1 | CW |
| DahongjunGeno62 | Lineage 1 | 4 | CW |
| DahongjunGeno63 | Lineage 1 | 5 | CW |
| DahongjunGeno39 | Lineage 2 | 2 | ML, JN |
| DahongjunGeno40 | Lineage 2 | 3 | DDG |
| DahongjunGeno41 | Lineage 2 | 1 | ML |
| DahongjunGeno42 | Lineage 2 | 1 | ML |
| DahongjunGeno47 | Lineage 2 | 1 | JN |
| DahongjunGeno48 | Lineage 2 | 1 | JN |
| DahongjunGeno49 | Lineage 2 | 1 | JN, DDG |
| DahongjunGeno50 | Lineage 2 | 1 | JN, DDG |
| DahongjunGeno52 | Lineage 2 | 2 | CW |
| DahongjunGeno55 | Lineage 3 | 1 | DDG |
| DahongjunGeno56 | Lineage 3 | 1 | DDG |
