# Supplementary figures and images for: Genetic Diversity of Dahongjun, the Commercially Important “Big Red Mushroom” from Southern China
Source: PLoS One. 2010 May 18;5(5):e10684. doi: 10.1371/journal.pone.0010684 (PMC2872671; doi:10.1371/journal.pone.0010684)

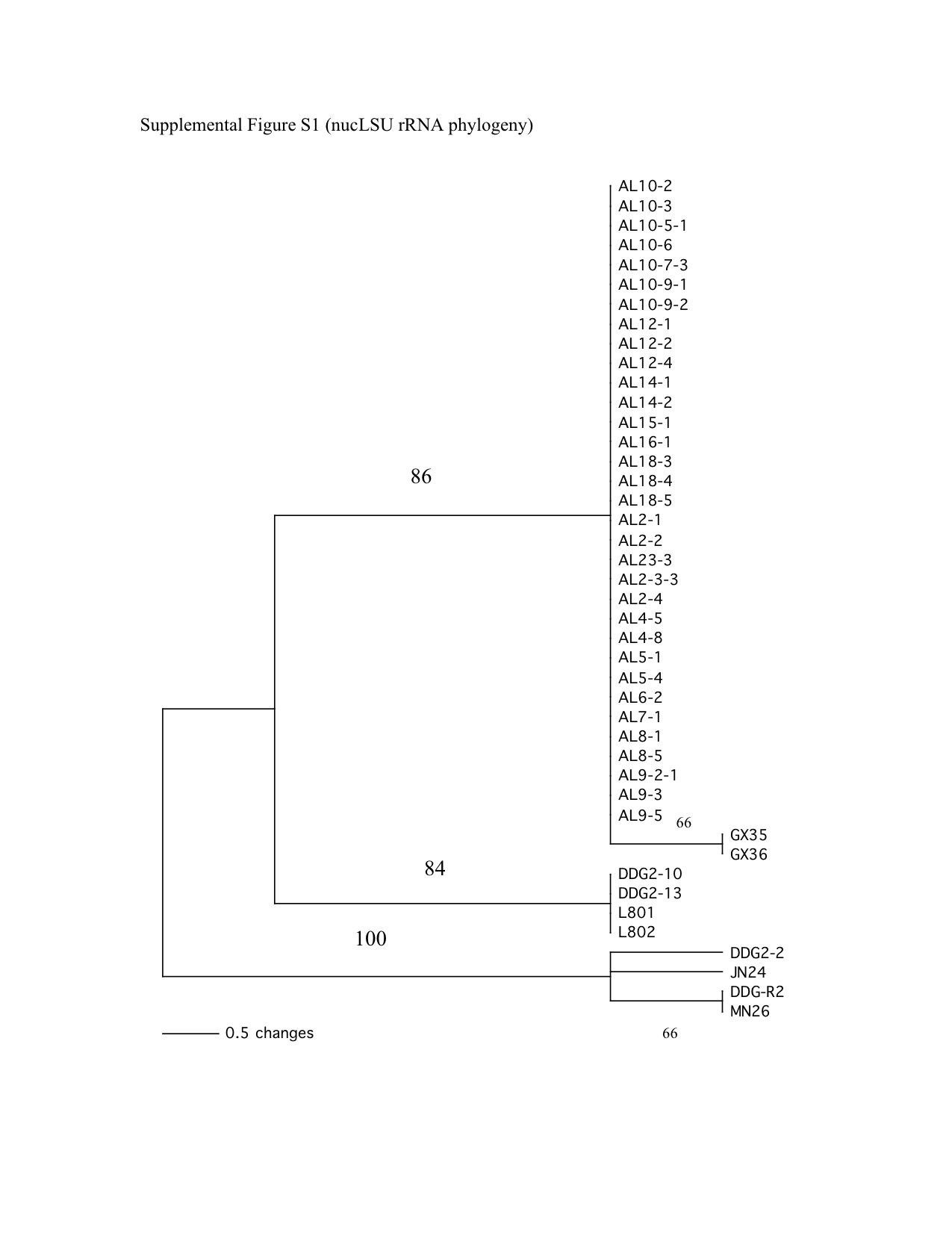

Supplement: Figure S1 — Maximum parsimony tree based on the nuclear large subunit ribosomal RNA (nucLSU rRNA) sequences from 43 representative isolates of Russula spp. collected from 5 study sites in 3 regions in southern China. Each strain is represented by its geographic affiliation (AL: Ailaoshan, central Yunnan; DDG: Dadugang, southern Yunnan; JN: Jinuo, southern Yunnan; ML: Mengla, southern Yunnan; and CW: Cangwu, eastern Guangxi) and one or more numbers representing our collection identification. Bootstrap support values (1000 replicates) are given above branches. Tree length = 15, Consistency index = 1, Retention index = 1. (0.16 MB TIF) [file pone.0010684.s003.tif]

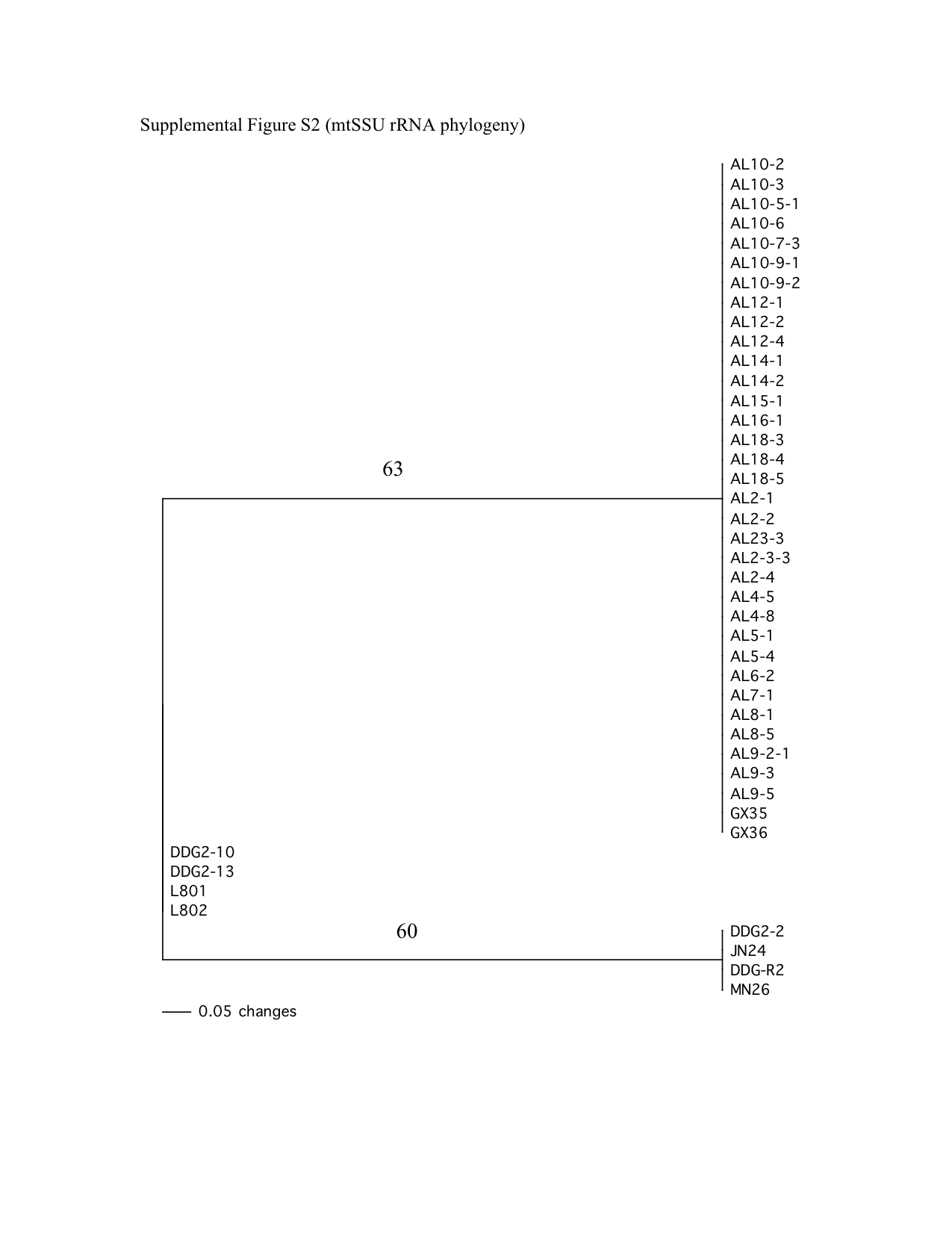

Supplement: Figure S2 — Maximum parsimony tree based on the mitochondrial small subunit ribosomal RNA (mtSSU rRNA) sequences from 43 representative isolates of Russula spp. collected from 5 study sites in 3 regions in southern China. Strain labels are identical to those in Figure S1. Bootstrap support values (1000 replicates) are given above branches. Tree length = 2, Consistency index = 1, Retention index = 1. (0.16 MB TIF) [file pone.0010684.s004.tif]

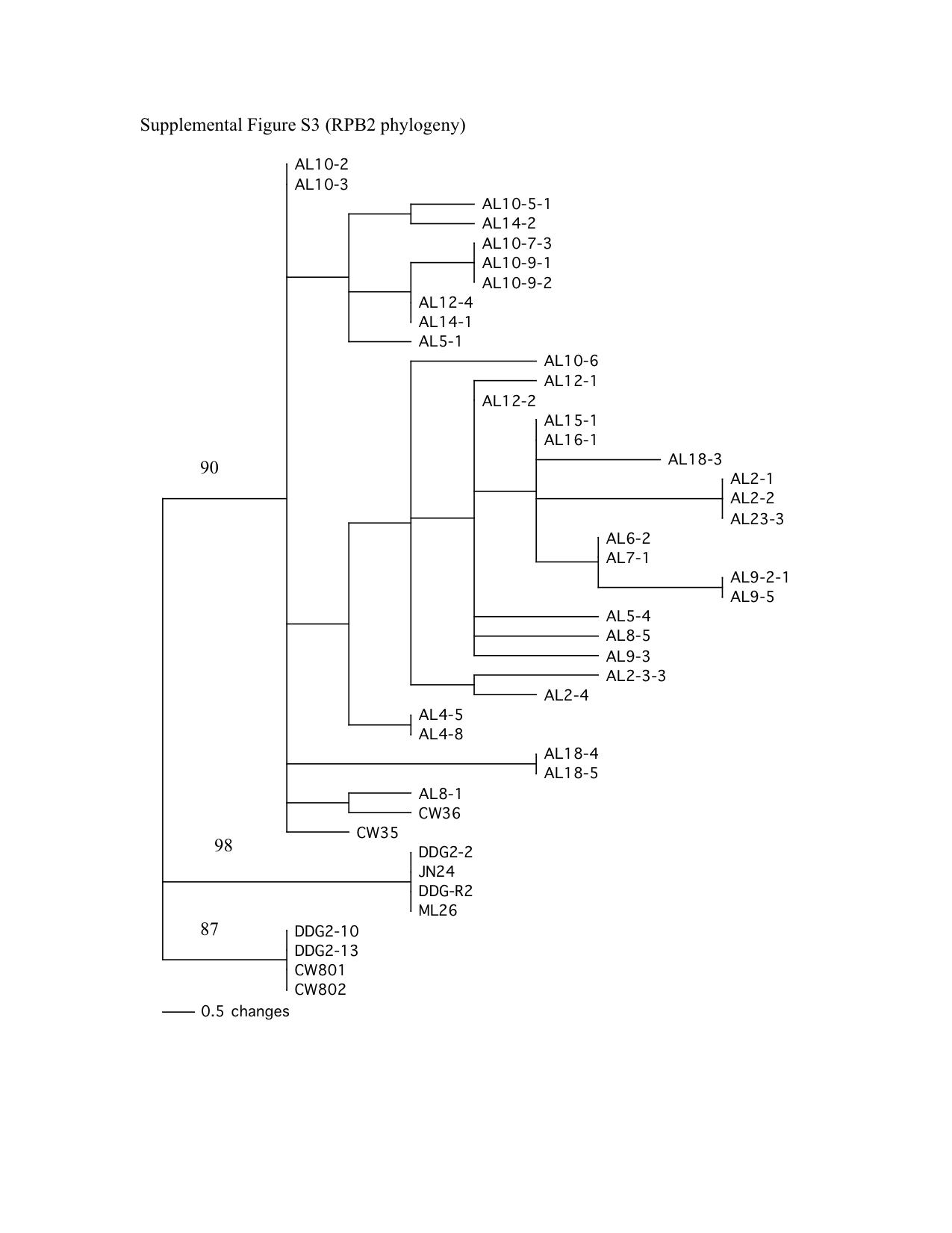

Supplement: Figure S3 — Maximum parsimony tree based on the nuclear RNA polymerase II large subunit (RPB2) from 43 representative isolates of Russula spp. collected from 5 study sites in 3 regions in southern China. Strain labels are identical to those in Figure S1. Bootstrap support values (1000 replicates) are given above branches. Tree length = 49, Consistency index = 0.673, Retention index = 0.853. (0.17 MB TIF) [file pone.0010684.s005.tif]

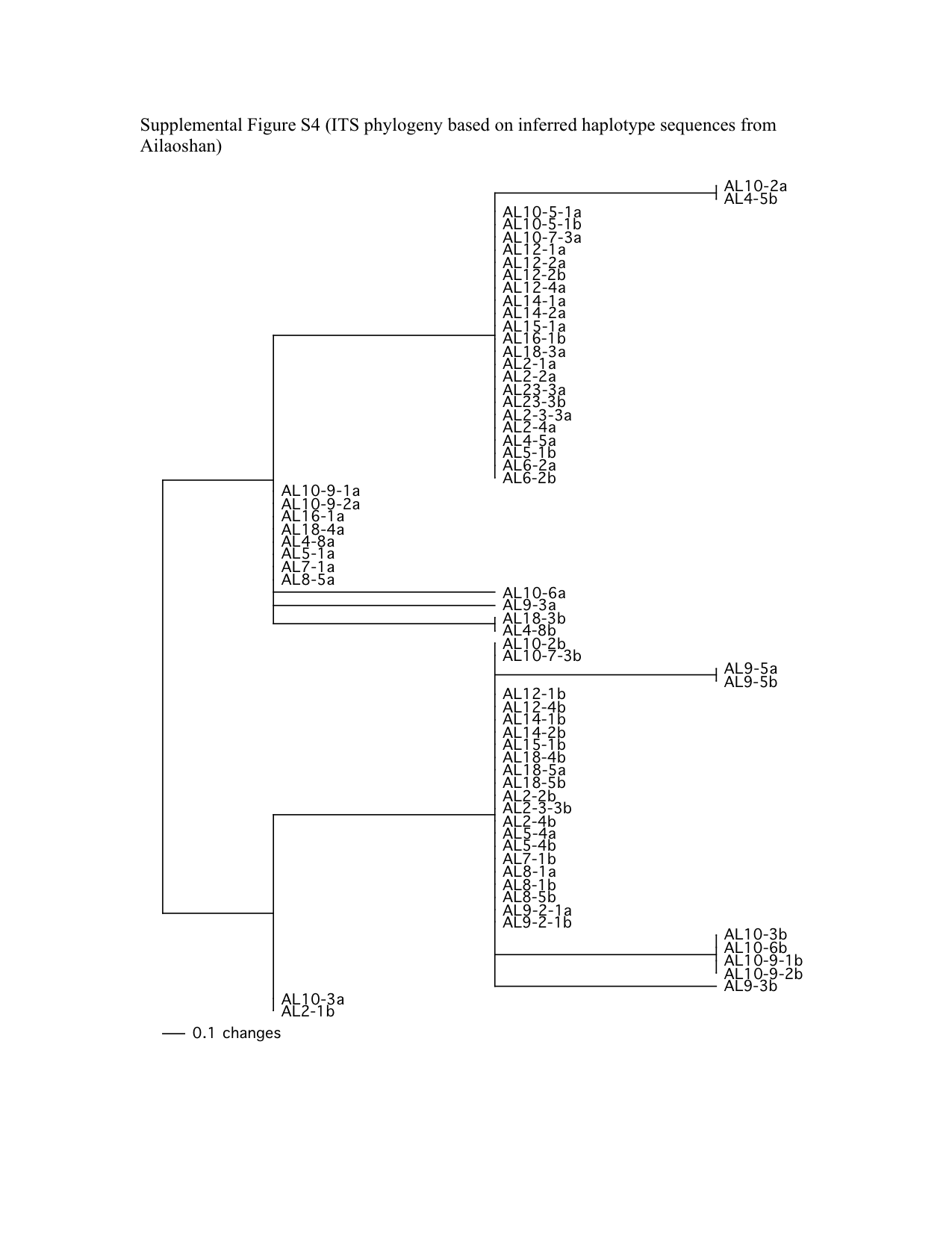

Supplement: Figure S4 — Maximum parsimony tree based on the inferred ITS haplotype sequences of 33 strains from Ailaoshan. Each ITS haplotype is represented by its geographic affiliation (AL: Ailaoshan, central Yunnan), one or more numbers representing our field collection identification, followed by a or b that represent the two alleles within an individual specimen. Tree length = 10, Consistency index = 0.6, Retention index = 0.955. (0.20 MB TIF) [file pone.0010684.s006.tif]

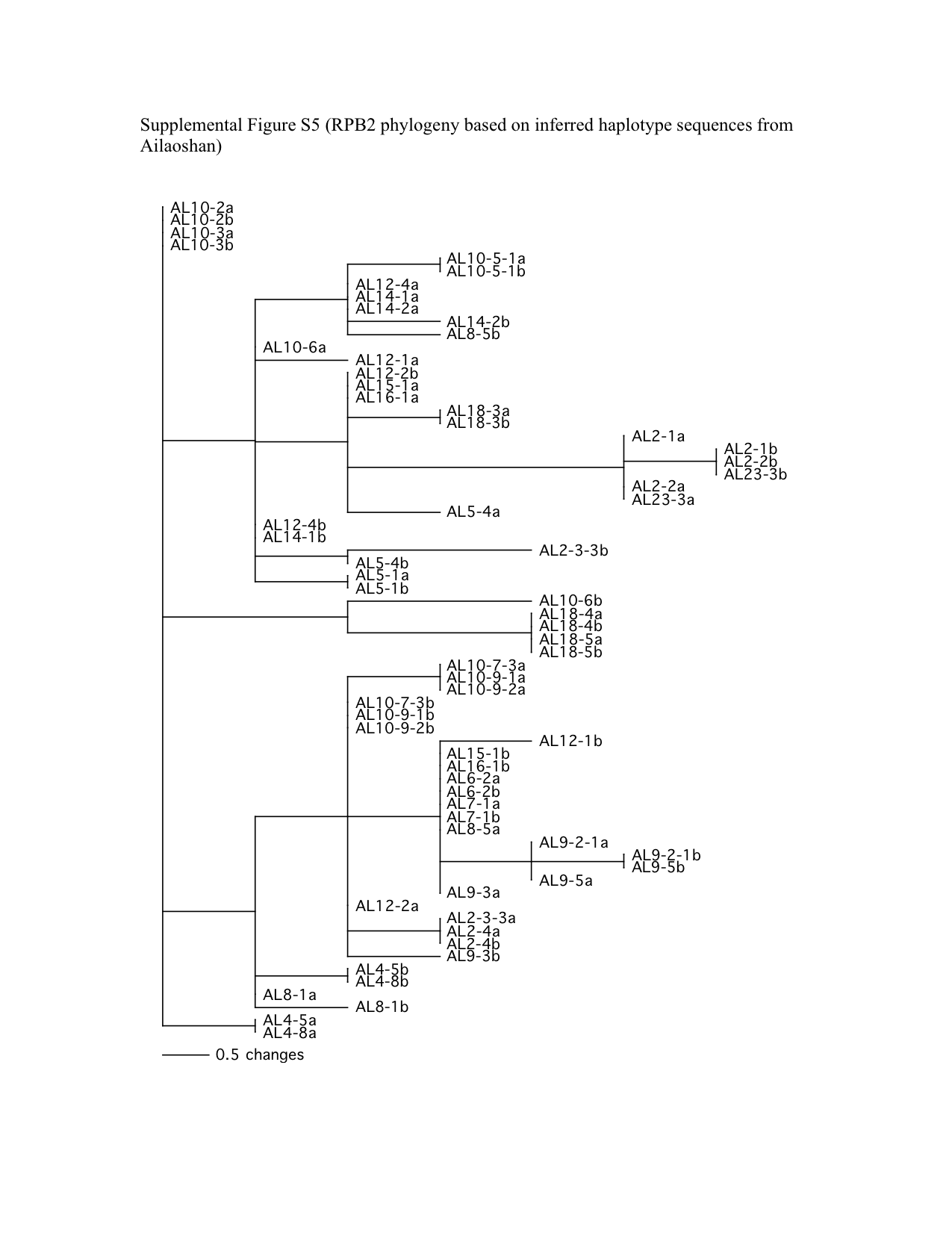

Supplement: Figure S5 — Maximum parsimony tree based on the inferred RPB2 haplotype sequences of 33 strains from Ailaoshan. Each RPB2 haplotype is represented by its geographic affiliation (AL: Ailaoshan, central Yunnan), one or more numbers representing our field collection identification, followed by a or b that represent the two alleles within an individual specimen. Tree length = 35, Consistency index = 0.486, Retention index = 0.860. (0.21 MB TIF) [file pone.0010684.s007.tif]
